# Transcirculation Approach for Mechanical Thrombectomy in Acute Ischemic Stroke: A Multicenter Study and Review of the Literature

**DOI:** 10.3389/fneur.2020.00347

**Published:** 2020-05-07

**Authors:** Jorge A. Roa, Alberto Maud, Pascal Jabbour, Guilherme Dabus, Avery Pazour, Sudeepta Dandapat, Santiago Ortega-Gutierrez, Diego Paez-Granda, Vladimir Kalousek, David M. Hasan, Edgar A. Samaniego

**Affiliations:** ^1^Department of Neurology, University of Iowa Hospitals and Clinics, Iowa City, IA, United States; ^2^Department of Neurology, Texas Tech University Health Sciences Center El Paso, El Paso, TX, United States; ^3^Department of Neurosurgery, Thomas Jefferson University Hospitals, Philadelphia, PA, United States; ^4^Department of Radiology, Miami Cardiac and Vascular Institute, Miami, FL, United States; ^5^Departments of Neurology, Neurosurgery and Radiology, University of Iowa Hospitals and Clinics, Iowa City, IA, United States; ^6^Department of Radiology, Virgen de la Arrixaca University Hospital, Murcia, Spain; ^7^Department of Neurology, University Clinical Hospital Center “Sestre Milosrdnice”, Zagreb, Croatia

**Keywords:** transcirculation approach, ischemic stroke, mechanical thrombectomy, neurointerventional technique, endovascular device, endovascular intervention

## Abstract

**Background:** Transcirculation approaches, which consist of primary catheterization of a target artery from the contralateral side or opposite cerebral circulation, provide alternate endovascular routes when anterograde interventions are not feasible. We aimed to assess the safety and efficacy of mechanical thrombectomy (MT) through a transcirculation route.

**Methods:** Six centers provided retrospective data on acute ischemic stroke (AIS) patients who underwent MT via transcirculation approaches. Demographics and technical details of the endovascular intervention were collected. Recanalization rates, peri-procedural complications and clinical/angiographic outcomes immediately after the procedure and at last available follow-up were assessed. A review of the literature reporting on AIS patients whom underwent transcirculation MT was also performed.

**Results:** Our multicenter study included 14 AIS patients treated through transcirculation routes. Mean age was 57.8 ± 11.9 years, and 10 (71.4%) were men. Mean NIHSS at admission was 18.4 (range 8–27). TICI 2b-3 recanalization was achieved in 10/14 (71.4%) patients. Three patients died after intervention: one due to late recanalization, one due to acute in-stent thrombosis, and one due to a procedure-related thromboembolic brainstem infarct. Of 11 surviving patients with follow-up available (mean 9.7 months), mRS 0–2 was achieved in 6 (54.5%) cases. Our review of the literature pooled a total of 37 transcirculation MT cases. Most common occlusions were tandem lesions (ICA + MCA = 64.9%) and BA + bilateral VA (18.9%). ACOM and PCOM were crossed in 18 (48.6%) cases each; one patient required a combined ACOM-PCOM approach. Primary recanalization technique included intra-arterial (IA) thrombolytics alone in 10 (27%), angioplasty ± stenting in 6 (16.2%), stent-retriever in 8 (21.6%), contact aspiration in 6, and combined (MT ± IA-thrombolytics) in 6 cases. Twenty-eight (75.7%) AIS patients achieved successful TIMI 2-3/TICI 2b-3 recanalization. After a mean follow-up of 6.7 months, 23/31 (74.2%) patients achieved a favorable functional outcome.

**Conclusions:** Transcirculation approaches may be used to access the target lesion when the parent artery cannot be crossed through conventional antegrade routes. These techniques are feasible but should be reserved as a bailout maneuver when anterograde MT is not possible. Newer endovascular devices have improved neurological and angiographic outcomes in transcirculation cases.

## Introduction

During the last decade, the treatment of acute ischemic stroke (AIS) has undergone a paradigm shift from a predominantly conservative approach to the use of endovascular mechanical thrombectomy (MT) to achieve recanalization of large vessel occluions (1). MT has shown significant benefits and has improved functional outcomes in AIS patients: number needed to treat = 2.6 (2). Although many of the earlier positive MT randomized trials had strict time windows up to 6 h, recent evidence supports a physiological rather than an exclusively time-based criteria to determine whether salvageable brain exists (3).

The development of more navigable endovascular devices has enabled safer access into distal lesions and enhanced the effectiveness of MT procedures (4). However, some cases display a complex vascular anatomy, with chronic inaccessible tandem occlusions (TO) involving proximal extra- and intracranial vessels. In this setting, traditional anterograde endovascular approaches through the parent vessel may be challenging. Retrograde or transcirculation approaches, which consist of primary catheterization of a target artery from the contralateral side or opposite cerebral circulation, can provide alternative pathways for successful recanalization of these cases.

The communicating artery crossed mainly depends on location of the lesion and presence of collaterals. The posterior communicating artery (PCOM) is used to approach lesions in the posterior circulation from the internal carotid arteries (ICAs, anterior-to-posterior) or lesions in the anterior circulation from the vertebral arteries (VAs, posterior-to-anterior). The anterior communicating artery (ACOM) and VAs provide appropriate left-to-right and right-to-left access. In rare occasions, the trigeminal artery has been used to access the posterior circulation (5). Our group previously described the use of transcirculation approaches for endovascular embolization of intracranial aneurysms, arteriovenous malformations, and dural fistulas (6).

With the advent of MT as standard of care, transcirculation approaches are increasingly used as a last resource when the conventional anterograde route is not feasible. Some of the most important drawbacks when using transcirculation techniques include increased procedural times (puncture to recanalization) and the potential risk of hemorrhagic complications or thromboembolic events in unaffected arterial territories. In this multicenter study, we present our experience in performing transcirculation MT with newer microcatheters, stent-retrievers, and aspiration devices. Additionally, a comprehensive review of the literature was conducted to evaluate the safety and efficacy of MT transcirculation approaches in patients with AIS.

## Methods

### Population and Data Collection

A multicenter collaborative database of subjects who underwent endovascular transcirculation interventions from September 2015 to April 2019 was generated and analyzed. The following centers participated: University of Iowa Hospitals and Clinics, IA, USA; Thomas Jefferson University Hospitals, PA, USA; Texas Tech University Health Sciences Center El Paso, TX, USA; Miami Cardiac and Vascular Institute, FL, USA; Virgen de la Arrixaca University Hospital, Murcia, Spain; and University Clinical Hospital Center “Sestre Milosrdnice,” Zagreb, Croatia. Each participating center screened their prospectively acquired database to identify MT cases performed via a transcirculation approach. Large vessel occlusion (LVO) was documented on CT angiography or MR angiography (MRA), and confirmed with digital subtraction angiography (DSA). Demographic information, clinical presentation, angioarchitecture, and technical details about the transcirculation procedure were collected. National Institute of Health Stroke Scale (NIHSS) score at admission, location of the occlusion, and thrombolysis in cerebral infarction (TICI) scores were also collected. Institutional review board approval was obtained at each center. Patient consent was waived given the retrospective and anonymous nature of the data analysis.

### Endovascular Technique

Technical details about the endovascular procedure were collected, including: arterial approach, communicating vessel crossed/direction, system used and primary recanalization technique. The indication for a transcirculation approach was presence of a refractory parent artery occlusion, defined as inability to cross a complete occlusion of the ipsilateral ICA or VA; thus, the only endovascular pathway to access the lesion was through a communicating artery from a different vascular territory.

### Post-procedural and Follow-Up Outcomes

A modified Rankin Scale (mRS) score ≤ 2 was defined as favorable functional outcome. TICI 2b-3 was considered a successful recanalization. Major complications were defined as periprocedural complications (24-h from MT) that resulted in significant morbidity (change in NIHSS ≥ 4) or mortality including: symptomatic stroke, hemodynamic instability, procedural halting, and death. Minor complications were defined as periprocedural complications that did not result in significant procedural morbidity or mortality. Clinically silent strokes were considered a minor complication.

### Review of the Literature

#### Eligibility Criteria

Following PRISMA guidelines, a comprehensive literature search was conducted in Ovid, MEDLINE, EMBASE, The Cochrane Library and Google Scholar databases. The search terms “transcirculation,” “retrograde,” “recanalization,” “endovascular,” “acute ischemic stroke,” “mechanical thrombectomy,” “circle of Willis,” and “contralateral approach” were used as main Medical Subject Headings. Only English-language articles published until July 2019 were included. Data extraction was performed independently by three different authors with cross verifications. No contact was established with the investigators for data verification.

#### Outcomes Definitions

For each study identified, location of the occlusion, communicating vessel crossed, MT technique, minor/major complications, and clinical/angiographic outcomes (both immediately after intervention and at follow-up) were assessed. Favorable clinical result was defined as mRS score ≤ 2, whereas successful angiographic outcome was considered as Thrombolysis in Myocardial Infarction (TIMI) 2-3 for older manuscripts or TICI 2b-3 for more recent reports.

### Statistical Analysis

Data are reported as mean ± standard deviation (SD) for continuous variables, and as frequency and percent for categorical variables. Aggregate average follow-up duration and its respective SD was calculated through the combination of variances. Aggregate statistics are presented as a frequency and percentage for categorical variables. Aggregate recanalization rates only included studies with angiographic follow-up available. Statistical analysis of data was performed using SPSS software (version 25.0 for Mac, IBM). No additional assessment for risk of bias across the studies was performed.

## Results

### Multicenter Study

#### Population

Fourteen AIS patients were treated endovascularly using transcirculation MT techniques (Table 1). Mean age was 57.8 ± 11.9 years (range 39–77 years), and 10 (71.4%) subjects were men. Mean NIHSS score at admission was 18.4 ± 6.8 points (range 8–27). Seven (53.8%) occlusions were located in the middle cerebral artery (MCA), 4 in the basilar artery (BA) and 3 in posterior cerebral artery (PCA) vascular territories.

**Table 1:** AIS patients treated via transcirculation approach.

***N***	**Age/sex**	**Location**	**NIHSS**	**Primary technique**	**Device**	**IA thrombolysis**	**Guide/catheter**	**Via**	**Discharge mRS/TICI**	**F/U**	**F/U mRS/TICI**
1	40s/W	R MCA	9	IA thrombolysis	N/A	15 mg IA-tPA	Neuron 53/Prowler LP	PCOM: P to A	2/2a	48 mo/DSA	0/3
2	50s/M	L MCA M2	8	IA thrombolysis	N/A	8 mg IA-tPA + Tirofiban	BGC 9F/Sofia 6F/SL-10	ACOM: R to L	2/2b	1 mo/–	1/–
3	40s/M	R MCA	27	Stent retrieval	Solitaire 4x40	None	BGC 9F/Sofia 6F/Velocity	ACOM: L to R	4/2a	3 mo/–	3/–
4	70s/W	R PCA	20	Contact aspiration	Penumbra 3MAX	None	Neuron MAX Penumbra ACE/3MAX	PCOM: A to P	6/3	Dead^*^	Dead^*^
5	70s/M	L MCA	17	Stent retrieval	Solitaire 6x20	None	Neuron MAX/Rebar	ACOM: R to L	4/0	12 mo/–	4/–
6	50s/M	L MCA	20	Contact aspiration	Penumbra 3MAX	None	Neuron MAX/Penumbra ACE/ 3MAX	ACOM: R to L	3/2b	3 mo/–	3/–
7	60s/M	L PCA	23	Contact aspiration	Penumbra 3MAX	None	Neuron MAX/Penumbra ACE/3MAX	PCOM: A to P	4/3	24 mo/–	4/–
8	50s/W	BA (R pons)	26	Contact aspiration and stent retrieval	Solitaire 4x20/Penumbra 3MAX	None	Neuron MAX/Cath React 68/Marksmann	PCOM: A to P	2/3	2 mo/–	2/–
9	40s/M	BA (R pons)	13	Stent retrieval/stenting	Solitaire 4x20/Stent Integrity 2.25x8	None	Envoy 6F/Echelon 14	PCOM: A to P	4/3	6 mo/DSA	3/3
10	30s/M	BA (L pons)	26	Contact aspiration	Penumbra 026	8 mg IA-tPA	Envoy 6F/Penumbra 026	PCOM: A to P	1/3	2 mo/–	0/-
11	60s/M	L MCA	8	Angioplasty stenting	Balloon Scepter XC 4x15/Stent Enterprise 4x39	N/A	Envoy 6F/Prowler LP	ACOM: R to L	1/0	3 mo/MRA	3/1 (ICH)
12	70s/M	R MCA	19	Contact aspiration and stent retrieval/stenting	Embotrap 5 33/Penumbra ACE 64/2 Stents Enterprise 4x16	None	Cello 9F/Headway Duo	ACOM: L to R	6/3	Dead^*^	Dead^*^
13	60s/M	R MCA	16	Contact aspiration and stent retrieval	Solitaire 4x40/Penumbra 3MAX	None	Neuron MAX/Penumbra ACE/Marksmann	PCOM: P to A	6/3	Dead^*^	Dead^*^
14	40s/W	R PCA	26	Contact aspiration and stent retrieval	Solitaire 6x40	None	Jet 7 Flex/Velocity	PCOM: A to P	3/2b	None	None

*A, anterior; ACOM, anterior communicating artery; AIS, acute ischemic stroke; BA, basilar artery; BGC, balloon guide catheter; F, French; F/U, follow-up; IA, intraarterial; ICA, internal carotid artery; ICH, intracranial hemorrhage; L, left; M, man; MCA, middle cerebral artery; mo, months; mRS, modified Rankin Scale; P, posterior; PA, parent artery; PCA, posterior cerebral artery; PCOM, posterior communicating artery; R, right; TICI, thrombolysis in cerebral infarction; tPA, tissue plasminogen activator; W, woman*.

* *Cause of death: subject 4 due to late recanalization; Subject 12 due to subtherapeutic anticoagulation; subject 13 due to brainstem infarct*.

#### Transcirculation Procedure

**Figure 1:**
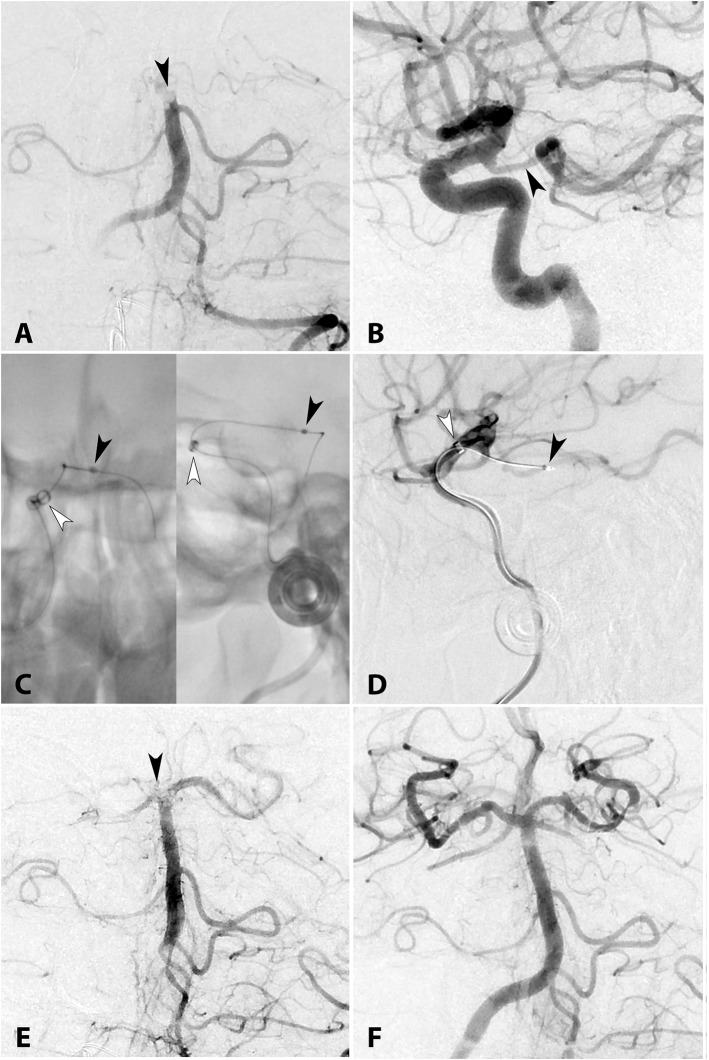
**(A)** AP diagnostic angiogram shows complete occlusion of the top of the BA (arrowhead). **(B)** Lateral right ICA angiogram demonstrates a wide patent PCOM (arrowhead). **(C)** Unsubstracted AP and lateral views and **(D)** lateral angiogram demonstrating the system used to navigate a microwire to the top of the BA occlusion. The intermediate catheter Cath React 68 (white arrowhead) is positioned at the origin of the PCOM and the microcatheter (black arrowhead) is navigated into the PCA. After a first pass of the stent-retriever (Solitaire Platinum 4 × 20 mm) under continuous manual suction through the intermediate catheter, AP angiography **(E)** shows a residual clot in the BA bifurcation occluding the right P1 (black arrowhead). **(F)** Seven passes were required to achieve complete recanalization. No residual thrombi were visualized in the anterior or posterior circulations. Total procedural time from skin puncture to revascularization was 233 min.

All patients required transcirculation intervention due to angiographic occlusion of the parent artery. Most cases were accessed via ICA catheterization (12/14, 78.6%), and 2 via VA catheterization. PCOM was crossed in 8 cases (6 anterior-to-posterior, 2 posterior-to-anterior) and ACOM in 6 cases (4 right-to-left, 2 left-to-right). MT was performed using stent-retriever alone in 3 cases (2 Solitaire [Medtronic] and 1 Embotrap [Cerenovus]), contact aspiration alone in 3 cases (3MAX catheter [Penumbra]), intra-arterial tissue plasminogen activator (IA-tPA) alone in 2 cases, combined stent-retriever plus aspiration in 4 cases (2 = Solitaire + 3MAX catheter, 1 = Solitaire + Penumbra ACE 64 catheter, 1 = Solitaire + Jet 7 Flex), combined aspiration plus intra-arterial thrombolytics in 1 case (Penumbra 026 reperfusion catheter + IA-tPA) and 1 angioplasty/stenting. Three (23.1%) patients underwent stenting due to the presence of an underlying plaque/stenosis: 2 Enterprise [Codman] and 1 Integrity [Medtronic]. Seven cases were performed using intermediate/aspiration catheters: 4 Penumbra ACE, 2 Sofia 6F [Microvention], and 1 React 68 [Medtronic]. The most common reperfusion catheter/microcatheters were Penumbra 3MAX in 3 cases, Marksman [Medtronic], Prowler LP Select [Cordis Neurovascular], and Velocity [Penumbra] in 2 cases each. A case depicting anterior-to-posterior approach via PCOM to perform MT in a patient with a BA occlusion is presented in Figure 1.

#### Procedural and Follow-Up Outcomes

**Figure 2:**
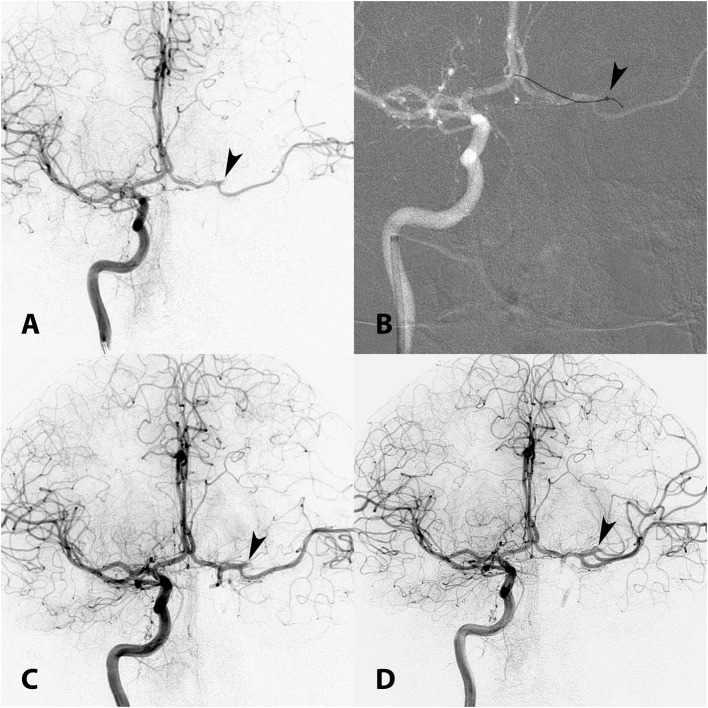
**(A)** AP angiogram shows occlusion of the superior branch of the left MCA (arrowhead). The ipsilateral left ICA is chronically occluded and the only way to access the target lesion is through the ACOM from the contralateral ICA. **(B)** Roadmap shows a Penumbra ACE intermediate catheter positioned in the high cervical right ICA and a 3MAX reperfusion catheter (arrowhead) positioned in the superior division of the left MCA. The 3MAX catheter was used to perform MT using contact aspiration via ACOM (right-to-left transcirculation approach). **(C)** First pass was unsuccessful. **(D)** Second pass achieved TICI 2b recanalization (arrowhead).

**Figure 3:**
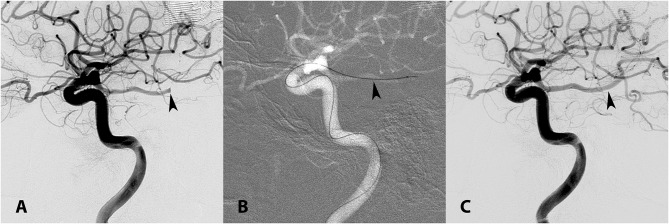
**(A)** Lateral left ICA angiogram demonstrating occlusion of the ipsilateral PCA (arrowhead). **(B)** A 3MAX reperfusion catheter (arrowhead) was used to perform contact aspiration via the left PCOM (anterior-to-posterior transcirculation approach). **(C)** TICI 3 recanalization was achieved (arrowhead) after one pass.

**Figure 4:**
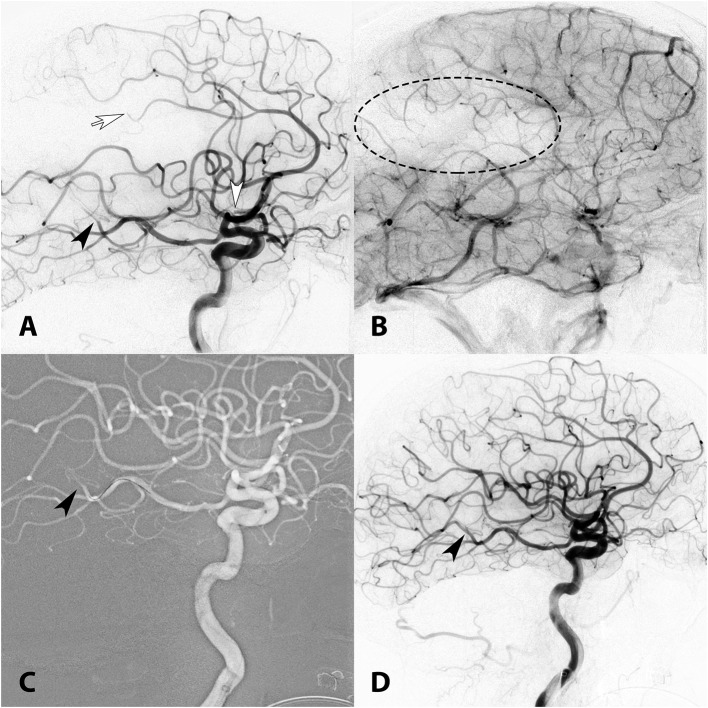
**(A)** Lateral right ICA angiogram showing multiple occlusions in the proximal MCA M2 (white arrowhead), distal pericallosal (white arrow), and distal PCA (black arrowhead). **(B)** A parenchymal defect is visualized in the late capillary phase of the angiogram (dotted ellipse). **(C)** A 3MAX reperfusion catheter was used to perform contact aspiration of the M2 and pericallosal occlusions and the PCA through PCOM (anterior-to-posterior transcirculation approach, arrowhead). **(D)** TICI 3 recanalization (arrowhead) post MT.

TICI 2b-3 recanalization was achieved in 10/14 (71.4%) patients (Figures 2–4). One arterial thrombus to another vascular territory was reported in a patient with a right MCA stroke that was fully recanalized (TICI 3) via PCOM, leading to a brainstem infarct due to occlusion of the BA. This patient died from the large stroke burden despite successful recanalization. Another patient with a right PCA occlusion fully recanalized via PCOM (anterior-to-posterior) approach died due to late recanalization and massive infarction. A third patient died due to subtherapeutic anticoagulation and concomitant in-stent thrombosis. Of 11 surviving patients with follow-up available (mean 9.7 months), mRS 0–2 was achieved in 6 (54.5%) cases.

### Review of the Literature

Our comprehensive review of the literature pooled a total of 12 studies describing 23 transcirculation MT cases (Table 2). Most common occlusion locations were MCA + tandem ICA occlusion (69.6%) and mid BA + bilateral VA occlusion (17.4%). ACOM and PCOM were crossed in 12 (52.2%) and 10 (43.5%) cases, respectively, 1 patient required combined ACOM-PCOM approach. Primary recanalization technique included IA-tPA alone in 8 (34.8%), stent-retriever in 5 (21.7%), angioplasty ± stenting in 5 cases, contact aspiration in 4 (17.4%), and combined (Solumbra ± IA-thrombolytics) in 1 case. Immediately after intervention, 18/23 (78.3%) AIS patients achieved successful TIMI 2-3/TICI 2b-3 recanalization. A total of 2 (8.7%) major procedure-related complications were reported. Available follow-up data included 20 clinical and 18 radiographic assessments. After a mean follow-up of 4.1 months, 18/20 (90%) patients achieved favorable functional outcomes (mRS 0–2), and 13/18 (72.2%) cases had successful recanalization.

**Table 2:** Case reports on endovascular recanalization via transcirculation approach in the literature.

**References**	**Cases**	**Location**	**Via**	**Treatment**	**Results**	**Complications**	**Length of F/U**	**F/U outcomes**
Ozdemir et al. (7)	8	MCA + tandem ICA (x8)	ACOM (x4), PCOM (x3), combined (x1)	IA-tPA (x8)	TIMI 3 (x2), TIMI 2 (x5), TIMI 1 (x1)	Small petechial asymptomatic hemorrhages (x3)	3 mo	mRS = 0–2 (x8), TIMI 2–3 (x5), TIMI 1 (x3)
Chiam et al. (8)	1	BA + bilateral VA	L ICA—PCOM	Angioplasty	30% residual BA stenosis^†^	None	2 weeks	No imaging, complete clinical improvement (mRS = 0)
Hui et al. (9)	1	R MCA + tandem ICA	L VA—PCOM	IA-tPA + CA	TIMI 2	Persistent R MCA thrombus (inferior division)	None	None
Liu et al. (10)	1	BA + bilateral VA	L ICA—PCOM	IA-tPA + CA	TIMI 3 in BA TIMI 2 in PCAs	Residual asymptomatic PCA thrombi	6 weeks	No imaging, complete clinical improvement (mRS = 0)
Padalino and Deshaies (11)	2	MCA + tandem ICA (x2)	ACOM	IA-abciximab + CA	TIMI 3 (x2)	Minimal asymptomatic in-stent stenosis (x1)	3–6 mo	mRS = 0–2 (x2), scattered asymptomatic distal infarcts (x1), symptomatic distal infarcts (x1)
Kim et al. (12)	1	R MCA + tandem ICA	R VA—PCOM	Stent retrieval	Full recanalization (TICI 3)	None	None	None
Nappini et al. (13)	1	L MCA + tandem ICA	R ICA—ACOM	Stenting	TICI 3	None	3 mo	Residual asymptomatic basal ganglia infarction, mRS = 0
Rossen et al. (14)	1	BA + bilateral VA	R ICA—PCOM	Stenting	TICI 3	None	6 mo	Wide BA patency, mRS = 2
Sultan-Qurraie et al. (15)	1	BA + bilateral VA	R ICA—PCOM	Stent retrieval	Recanalized BA	None	12 mo	Bilateral (right>left) asymptomatic cerebellar infarctions, mRS = 1
Perez-Montilla et al. (16)	1	R ICA terminus (thrombosed giant aneurysm)	L ICA—ACOM	Stenting	Successful recanalization	None	12 mo	Giant aneurysm recanalized at 4 mo, successful embolization; mRS = 1
Amuluru et al. (17)	2	MCA + tandem ICA (x2)	ACOM	Stent retrieval	TICI 2a (x2)	None	1 mo	Scatered asymptomatic infarctions (x2), mRS = 0–2 (x2)
Grossberg et al. (18)	3	L MCA + tandem ICA	PCOM	Stent retrieval	TICI 2a	None	Death	Death (delayed intervention, cerebral edema)
		R ACA + tandem ICA	L ICA—ACOM	Combined (IA-tPA + Solumbra)	TICI 3	ECA thrombus 48 h later, treated with IA-abciximab + oral clopidogrel	3 mo	Scattered ACA and distal MCA infarcts, mRS = 3
		R ICA terminus	L ICA—ACOM	Stent retrieval	Recanalized, hemorrhagic transformation	Large ICH in the MCA territory 48 h later, required hemicraniectomy	3 mo	Persistent large R MCA-ACA stroke, mRS = 4
Aggregate Statistics	23	ICA terminus (x2) ACA + tandem ICA (x1) MCA + tandem ICA (x16) BA + bilateral VA (x4)	ACOM (x12) PCOM (x10) Combined (x1)	IA-tPA (x8) Angioplasty ± Stenting (x5) CA ± IA-TL (x4) Stent retrieval (x5) Combined (x1)	-TIMI 2-3/TICI 2b-3 = 18 (78.3%) - TIMI 1/TICI ≤ 2a = 5 (21.7%)	-Minor (asymptomatic) = 6 - Major (symptomatic/procedure-related death) = 2	Mean 4.1 mo	- Deaths = 1 - Clinical F/U (= 20), mRS 0–2 = 18 (90%) -Angiographic F/U (*n* = 18), successful recanalizatio*n* (asymptomatic infarcts) = 13 (72.2%)
Roa et al. (2019)^*^	14	MCA (x8) BA (x3) PCA (x3)	PCOM (x8) ACOM (x6)	Stenting (x1), IA-tPA (x2), Stent retrieval (x3), CA (x3), Combined (x5)	Failed recanalization (x2), TICI 2a (x2), TICI 2b-3 (x10)	Arterial thromboembolism (x1), major in-stent stenosis (x1)	Mean 10.4 mo	-Deaths = 3 - Clinical F/U (*n* = 10), mRS 0–2 (x4), mRS 3–5 (x6) - Angiographic F/U (*n* = 3): TICI 3 (x2), TICI I (x1)

*ACA, anterior cerebral artery; ACOM, anterior communicating artery; AIS, acute ischemic stroke; BA, basilar artery; CA, contact aspiration; ECA, external carotid artery; F/U, follow-up; IA, intra-arterial; ICA, internal carotid artery; MCA, middle cerebral artery; mRS, modified Rankin scale; mo, months; PCA, posterior cerebral artery; PCOM, posterior communicating artery; SAC, stent-assisted coiling; TICI, thrombolysis in cerebral infarction; TIMI, thrombolysis in myocardial infarction; TL, thrombolytics; tPA, intra-arterial tissue plasminogen activator; VA, vertebral artery*.

* *Refers to the cohort described in the current study (not included in the aggregate statistics, showed for comparison)*.

*Considered TIMI 1 for aggregate statistics*.

## Discussion

MT via transcirculation approaches in AIS patients is feasible. These techniques should be attempted as a bailout maneuver in patients with complex angioarchitectures and refractory recanalization of the parent artery. Conventional anterograde endovascular routes should be the first option.

### Transcirculation Technique in Acute Ischemic Stroke

Transcirculation approaches in the treatment of aneurysms, arteriovenous malformations and arteriovenous fistulas have been described extensively (6). Treatment of AIS through MT transcirculation approaches is limited to case reports (Table 2). Grossberg et al. (18) reported three patients treated with transcirculation MT using endovascular devices. In two cases, a smaller stent-retriever (3 mm diameter) Trevo XP [Stryker] was used to withdraw clot across ACOM and PCOM. In the third case, the 3MAX reperfusion catheter was used to achieve recanalization of the anterior cerebral artery through ACOM. In our series, a variety of devices and techniques were used to achieve recanalization, these included stent-retrievers of different diameters (Solitaire [Medtronic] 4 × 20, 4 × 40, 6 × 20 and 6 × 40 mm; and Embotrap II [Cerenovus] 5 × 33-mm) as well as contact aspiration catheters, mainly the 3MAX reperfusion catheter (21.4% of cases). The 3MAX reperfusion catheter has been in the frontline of contact aspiration of M2 and M3 occlusions (19). It has a distal 0.035″ inner diameter and a 153 cm length, enabling excellent support, and distal navigation. It can be used for contact aspiration and to support catheterization with ACE 64 reperfusion catheters. Other microcatheters with excellent tractability and compliance used in this series are the Marksman [Medtronic] and Prowler LP Select [Cordis Neurovascular]. Another key technical development in performing distal MT with complex anatomies is the use of compliant support/reperfusion catheters, such as those used in these case series (Penumbra ACE and Sofia 6F). Of note, 8 procedures (57.1%) were performed only with aspiration catheters: Cath React 68 = 1, Jet 7 Flex = 1, Penumbra ACE = 4, Sofia 6F = 2.

### Technical Nuances of Transcirculation Procedures

One of the drawbacks of attempting a transcirculation MT is that these procedures are longer than anterograde approaches. Due to heterogeneity among studies, we were not able to calculate aggregate statistics regarding time from puncture to recanalization. The first pass effect (FPE), defined as achieving complete recanalization with a single thrombectomy device pass, is an important predictor of good clinical outcome after MT. An analysis of 354 AIS patients treated with MT from the NASA registry showed that mRS score ≤ 2 was significantly higher in FPE (*n* = 89) vs. non-FPE (*n* = 265) groups (61.3 vs. 35.3%, OR 1.7, *P* = 0.013). Moreover, independent predictors of achieving FPE in this cohort were use of balloon guide catheters (BGCs) and non-ICA terminus occlusion (20). The inherent difficulty to access the target lesion through a conventional antegrade approach, leads to the use of an alternative transcirculation MT, thus leading to increased procedural times and less FPE. The comprehensive literature review showed a technical success rate (TIMI 2-3/TICI 2b-3) of 78.3%; and good functional results at follow-up: mRS 0–2 = 90%; our multicenter study showed poorer functional outcomes: mRS 0–2 = 40%. This difference is probably related to the different methods used in each study. Most studies published in the literature and included in our review were technical notes or case reports (Table 2). This encompasses an intrinsic bias that favors reporting cases with good clinical outcomes, which may not accurately represent current endovascular MT transcirculation results. In our case series the transcirculation approach was used as a last resource when the anterograde route was not feasible. This patient population will have worse outcomes due to complex anatomies and longer times to achieve recanalization.

Transcirculation techniques require navigation across acute angles: the angle formed between the PCOM and ipsilateral P1, or the angle between the supraclinoid ICA-anterior cerebral artery (ICA/A1) junction in trans-ACOM access. When catheterizing contralateral lesions through the Circle of Willis, these narrow angles may pose increased tension over the triaxial system, making navigation more difficult and increasing the risk of iatrogenic arterial dissection. Moreover, the assessment of tensions through the system at the time of MT is limited, as the intermediate catheter is positioned proximally and far from the target lesion.

An analysis of 866 AIS patients whom underwent endovascular treatment over 12 years found iatrogenic arterial dissection to be a rare complication (*n* = 18. 2%). However, all dissections occurred in patients treated with MT: 10 (55.6%) contact aspiration, 4 (22.2%) stent-retriever, and 4 stenting/angioplasty (21). Catheterization of small ACOMs and PCOMs can significantly alter their trajectory and angulation (22). Although we did not encounter arterial dissections in this study, one must consider the caliber of the communicating vessels and the tension over the bi/triaxial system during transcirculation MT to avoid this uncommon but potentially fatal complication.

Another major concern with treatment of AIS from an unaffected arterial territory is the risk of thromboembolic events while attempting to recanalize a distal occlusion. Our literature review of transcirculation MT procedures demonstrated that most of these procedural thromboembolic complications are asymptomatic. One patient in our series developed a posterior circulation stroke as a result of a transcirculation MT performed in the anterior circulation through the PCOM.

An excellent technique to avoid distal embolization of thrombi fragments during MT is the use of BGCs. While flow arrest may be beneficial in the clot retrieval process, flow reversal may constitute the most important aspect of the technique (23). BGCs may be used in transcirculation MT when withdrawing the stent-retriever across ACOM or PCOM. However, it is unclear if the benefits of using BGCs can also be obtained in transcirculation procedures. In our cohort, no procedure was performed with a BGC. On a case-by-case basis, stroke severity has to justify MT through an unaffected vascular territory.

All transcirculation MT procedures in this study were performed due to parent artery occlusion. It has been demonstrated that clinical outcomes of AIS patients with diffuse atherosclerotic disease and concurrent large intra- and extracranial artery occlusion are relatively worse compared with intracranial one-vessel-occlusion (24). Most of these patients have chronic occlusions, which pose a challenge for an anterograde endovascular approach. As an alternative to a transcirculation MT, an antegrade recanalization of chronically occluded ICAs may be attempted. Our group has developed a classification system and described the endovascular/hybrid technique to cross these chronically occluded lesions (25–27). A selected group of patients with a completely occluded ICA (no visualization of a stump at the origin of the ICA and without supraclinoid reconstitution through collateral flow from the external carotid artery); may not benefit from any type of anterograde intervention (28). In these patients, a transcirculation MT may be the only option.

### Limitations

The main limitations are that this is a retrospective study with a small sample size. We also do not report time to puncture or time to recanalization; both variables are important success factors at the time of MT. The main goal of this study is to report the technical nuances of an approach that is rarely used in MT.

## Conclusion

MT via transcirculation approaches is feasible. These techniques should be attempted as a bailout maneuver in patients with refractory parent artery occlusions that are not amenable to recanalization despite conventional anterograde endovascular routes. The development of newer endovascular catheters and MT devices has eased the access of distal target lesion in patients with unfavorable angioarchitecture.

## Data Availability Statement

The datasets generated for this study are available on request to the corresponding author.

## Ethics Statement

The studies involving human participants were reviewed and approved by the University of Iowa HawkIRB. Written informed consent for participation was not required for this study in accordance with the national legislation and the institutional requirements. Written informed consent was not obtained from the individual(s) for the publication of any potentially identifiable images or data included in this article.

## Author Contributions

ES: study design. ES and JR: acquisition of data, data analysis, and preparation of the manuscript. All other authors: substantially contributed with acquisition of data. SO-G and DH: critical review of the manuscript.

## Conflict of Interest

SO-G is a consultant for Stryker Neurovascular and Medtronic. GD is a consultant for Medtronic, Microvention, Penumbra, and Cerenovus. PJ and ES are consultants for Medtronic and Microvention. The remaining authors declare that the research was conducted in the absence of any commercial or financial relationships that could be construed as a potential conflict of interest. The handling editor declared a past co-authorship with several of the authors GD and SO-G.

## References

[1] Josh K Chen M Chapter 3: Indications for mechanical thrombectomy. In: Samaniego EA, Hasan D, editors. Acute Stroke Management in the Era of Thrombectomy. 1st ed Springer Nature: Switzerland AG (2019). p. 25–37

[2] Goyal M Menon BK van Zwam WH Dippel DW Mitchell PJ Demchuk AM. Endovascular thrombectomy after large-vessel ischaemic stroke: a meta-analysis of individual patient data from five randomised trials. Lancet. (2016) 387:1723–31. 10.1016/S0140-6736(16)00163-X 26898852

[3] Jovin TG Liebeskind DS Gupta R Rymer M Rai A Zaidat OO. Imaging-based endovascular therapy for acute ischemic stroke due to proximal intracranial anterior circulation occlusion treated beyond 8 hours from time last seen well: retrospective multicenter analysis of 237 consecutive patients. Stroke. (2011) 42:2206–11. 10.1161/STROKEAHA.110.604223 21778444

[4] Samaniego EA Roa JA Limaye K Adams HP Jr. Mechanical thrombectomy: emerging technologies and techniques. J Stroke Cerebrovasc Dis. (2018) 27:2555–71. 10.1016/j.jstrokecerebrovasdis.2018.05.025 29960666

[5] Mulder M Lycklama ANGJ Dinkelaar W de Rooij T van Es A van der Kallen BF. Thrombectomy in posterior circulation stroke through persistent primitive trigeminal artery: a case report. Interv Neuroradiol. (2015) 21:715–8. 10.1177/1591019915609122 26464287 PMC4757357

[6] Roa JA Ortega-Gutierrez S Martinez-Galdamez M Maud A Dabus G Pazour A. Transcirculation approach for endovascular embolization of intracranial aneurysms, arteriovenous malformations, and dural fistulas: a multicenter study. World Neurosurg. (2020) 134:e1015–e27. 10.1016/j.wneu.2019.11.078 31759150

[7] Ozdemir O Bussiere M Leung A Gulka I Lee D Chan R. Intra-arterial thrombolysis of occluded middle cerebral artery by use of collateral pathways in patients with tandem cervical carotid artery/middle cerebral artery occlusion. AJNR Am J Neuroradiol. (2008) 29:1596–600. 10.3174/ajnr.A1163 18524975 PMC8119044

[8] Chiam PT Mocco J Samuelson RM Siddiqui AH Hopkins LN Levy EI. Retrograde angioplasty for basilar artery stenosis: bypassing bilateral vertebral artery occlusions. J Neurosurg. (2009) 110:427–30. 10.3171/2008.7.JNS08436 19012478

[9] Hui FK Narayanan S Cawley CM. Posterior-to-anterior circulation access using the penumbra stroke system for mechanical thrombectomy of a right middle cerebral artery thrombus. World Neurosurg. (2010) 73:17–21. 10.1016/j.surneu.2009.05.020 20452865

[10] Liu W Kung DK Mahaney KB Rossen JD Jabbour PM Hasan DM. Anterior-to-posterior circulation approach for mechanical thrombectomy of an acutely occluded basilar artery using the penumbra aspiration system. World Neurosurg. (2012) 77:398 E17–20. 10.1016/j.wneu.2011.04.025 22120391

[11] Padalino DJ Deshaies EM. Tandem middle cerebral artery-internal carotid artery occlusions: reduced occlusion-to-revascularization time using a trans-anterior communicating artery approach with a penumbra device. J Neurosurg. (2012) 116:665–71. 10.3171/2011.10.JNS111516 22196094

[12] Kim SK Baek BH Heo TW Yoon W. Successful cross-circulation stent-retriever embolectomy through posterior communicating artery for acute MCA occlusion by using trevo XP provue. Neurointervention. (2016) 11:55–8. 10.5469/neuroint.2016.11.1.55 26958415 PMC4781920

[13] Nappini S Limbucci N Leone G Wlderk A Mangiafico S. Trans-anterior communicating artery primary stenting in acute tandem middle cerebral artery-internal carotid artery occlusion due to thoracic aortic stent graft. World Neurosurg. (2017) 106:1050.e21–50.e24. 10.1016/j.wneu.2017.06.187 28710049

[14] Rossen JD Samaniego EA Paullus M Ortega-Gutierrez S. Hybrid retrograde-antegrade recanalization of acute basilar artery occlusion. Interv Neurol. (2017) 6:263–7. 10.1159/000479704 29118804 PMC5662970

[15] Sultan-Qurraie A Rozansky G Cox JA Lazzaro M. Cross-circulation thrombectomy with use of a stent retriever: a case report. Interv Neuroradiol. (2017) 23:422–6. 10.1177/1591019917706191 28480772 PMC5684909

[16] Perez-Montilla ME Bravo Rey IM Bautista Rodriguez MD Alvarado SV Bravo-Rodríguez FA Delgado Acosta. Acute occlusion of a giant aneurysm of the internal carotid artery: recanalisation of the middle cerebral artery through the contralateral carotid artery. Neurologia. (2017) 32:480–4. 10.1016/j.nrleng.2015.11.013 26774414

[17] Amuluru K Romero CE Pyle L El-Ghanem M Al-Mufti F. Mechanical thrombectomy of acute middle cerebral artery occlusion using trans-anterior communicating artery approach. World Neurosurg. (2018) 112:46–52. 10.1016/j.wneu.2018.01.038 29339323

[18] Grossberg JA Haussen DC Bouslama M Nogueira RG. Stent-retriever thrombectomy across circle of willis. World Neurosurg. (2018) 115:47–53. 10.1016/j.wneu.2018.03.212 29631079

[19] Altenbernd J Kuhnt O Hennigs S Hilker R Loehr C. Frontline ADAPT therapy to treat patients with symptomatic M2 and M3 occlusions in acute ischemic stroke: initial experience with the penumbra ACE and 3MAX reperfusion system. J Neurointerv Surg. (2018) 10:434–9. 10.1136/neurintsurg-2017-013233 28821628 PMC5909737

[20] Zaidat OO Castonguay AC Linfante I Gupta R Martin CO Holloway WE. First pass effect: a new measure for stroke thrombectomy devices. Stroke. (2018) 49:660–6. 10.1161/STROKEAHA.117.020315 29459390

[21] Goeggel Simonetti B Hulliger J Mathier E Jung S Fischer U Sarikaya H. Iatrogenic vessel dissection in endovascular treatment of acute ischemic stroke. Clin Neuroradiol. (2019) 29:143–51. 10.1007/s00062-017-0639-z 29098320 PMC6394531

[22] Blackburn SL Kadkhodayan Y Shekhtman E Derdeyn CP Cross DT III Moran CJ. Treatment of basilar tip aneurysms with horizontal PCA to PCA stent-assisted coiling: case series. J Neurointerv Surg. (2013) 5:212–6. 10.1136/neurintsurg-2012-010301 22453336

[23] Nguyen TN Malisch T Castonguay AC Gupta R Sun CH Martin CO Balloon guide catheter improves revascularization and clinical outcomes with the Solitaire device: analysis of the North American solitaire acute stroke registry. Stroke. (2014) 45:141–5. 10.1161/STROKEAHA.114.004828 24302483

[24] Assis Z Menon BK Goyal M Demchuk AM Shankar J Rempel JL. Acute ischemic stroke with tandem lesions: technical endovascular management and clinical outcomes from the ESCAPE trial. J Neurointerv Surg. (2018) 10:429–33. 10.1136/neurintsurg-2017-013316 29021311

[25] Hasan D Zanaty M Starke RM Atallah E Chalouhi N Jabbour P Feasibility, safety, and changes in systolic blood pressure associated with endovascular revascularization of symptomatic and chronically occluded cervical internal carotid artery using a newly suggested radiographic classification of chronically occluded cervical internal carotid artery: pilot study. J Neurosurg. (2018) 1:1–10. 10.3171/2019.1.JNS183337 29775153

[26] Zanaty M Howard S Roa JA Alvarez CM Kung DK McCarthy DJ Cognitive and cerebral hemodynamic effects of endovascular recanalization of chronically occluded cervical internal carotid artery: single-center study and review of the literature. J Neurosurg. (2019) 29:1–9. 10.1016/j.wneu.2018.09.230 30925474

[27] Zanaty M Samaniego EA Teferi N Kung DK Nakagawa D Hudson J. Hybrid surgery for internal carotid artery revascularization. World Neurosurg. (2019) 121:137–44. 10.1016/j.wneu.2018.09.230 30312821

[28] Zanaty M Roa JA Jabbour PM Samaniego EA Hasan DM. Recanalization of the chronically occluded internal carotid artery: review of the literature. World Neurosurg X. (2019) 5:100067. 10.1016/j.wnsx.2019.100067 31872191 PMC6920090

